# A Critical Review of Post-Childbirth Pain Experiences and Management in Relation to Postpartum Depression Risk for Racial and Ethnic Minorities

**DOI:** 10.1007/s11920-025-01655-z

**Published:** 2025-12-22

**Authors:** Sandraluz Lara-Cinisomo, Sudhamshi Beeram, Melany E. Romero

**Affiliations:** Department of Health and Kinesiology, University of Illinois Urbana-Champaign, 1206 S. Fourth Street, Champaign, IL USA

**Keywords:** Prenatal pain, Postpartum pain, Childbirth experiences, Pain management, Risk factors

## Abstract

**Purpose:**

This critical review examines literature published between January 2020 and January 2025, focusing on overlapping pain-related factors during and after childbirth (e.g., pain experiences and management). These factors may increase vulnerability to postpartum depression, especially among racial and ethnic minorities.

**Recent Findings:**

The findings from the 23 studies reviewed indicate that several factors contribute to peripartum pain experienced by individuals giving birth. Factors influencing the birthing person’s pain experiences include their mental health during pregnancy (such as depression and anxiety), delivery method (especially cesarean), pain management practices, discrimination toward racial and ethnic minorities, and overall neglect of pain. Additionally, healthcare providers’ beliefs about pain management play a role in postpartum pain experiences.

**Summary:**

Individuals with a history of depression or anxiety often experience more severe postpartum pain. Mode of delivery is an important factor, as cesarean deliveries are associated with more severe pain than vaginal deliveries. However, intrapartum experiences and pain management significantly influence pain ratings. Analgesics during and after labor may buffer postpartum pain, but not always. Additionally, pain relief medications for patients with opioid use disorders can impact postpartum pain management. While healthcare providers rely on clinical assessments and patient-centered approaches to inform postpartum pain management, data from racial and ethnic minorities revealed that healthcare professionals often fail to recognize these patients' pain. This underscores the disparities in perspectives and experiences among patients. Post-childbirth pain experiences and their management strategies may increase the risk of postpartum depression, highlighting the necessity for researchers and practitioners to consider them.

## Introduction

Postpartum depression (PPD) is a significant public health issue that affects approximately 10% to 20% of women in the U.S [1]. A study indicated that rates of postpartum depression (PPD) increased significantly between 2010 and 2021 for all racial and ethnic groups, with the largest increase observed among racial and ethnic minorities over time [2]. Pain and inadequate pain management during and after childbirth are known risk factors for PPD. Pain serves as a stressor that triggers numerous physiological changes in response to an unpleasant stimulus. While acute pain responses can facilitate recovery [3], prolonged exposure to pain can disrupt homeostasis and negatively impact mental health, including increasing the risk for PPD [4].

Several risk factors associated with postpartum pain may also contribute to PPD, such as experiencing pain before and during pregnancy [5]. Individuals who give birth are at a higher risk of chronic pain if not effectively managed, particularly those who undergo cesarean deliveries [6, 7]. Psychosocial factors, including racial discrimination, further increase the risk of PPD [8]. This is particularly concerning as Black and Latina individuals are often less likely to receive appropriate postpartum pain management [9, 10]. Despite the existing disparities and shared risk factors between postpartum pain and PPD, research focusing on minority birthing individuals is limited.

There is a critical need to understand the pain-related factors that may contribute to PPD, as both conditions can be burdensome for the birthing person, their families, and society. Pain after childbirth and depression frequently co-occur. Additionally, individuals at risk of experiencing persistent pain after childbirth may also face related risk factors for PPD, such as racial discrimination. By identifying post-childbirth pain and management strategies that may increase psychological vulnerability, particularly among racial and ethnic minorities, we can develop targeted interventions to address overlapping risk factors.

While there is empirical evidence linking pain to depression, the complex experiences of racial and ethnic minority birthing individuals have yet to be comprehensively examined. This critical review addresses this gap by focusing on pain-related factors that elevate the risk of PPD, such as individual, psychosocial, and structural elements.

## Methods

Two authors conducted the literature searches using the databases PubMed, PsycINFO, ScienceDirect, Scopus, and Cochrane to extract articles written in English from 2020 to 2025. A comprehensive list of keywords was developed to guide the search process (see Table 1). Studies were included if they were peer-reviewed, published between January 2020 and January 2025, written in English, conducted in the United States, and investigated pain during or after childbirth.

**Table 1:** Summary of search terms and strategy

Category	Search Terms/Strategy
Core Search terms	“pain” AND (“prenatal” OR “postpartum” OR “perinatal”) AND “depression”
Additional keywords (added with AND)	management, labor, risk, cultural, report, differences, beliefs, care, analgesic, delivery, medication, practice, Black, Asian, minority, immigrant, intervention, Hispanic, postpartum period, discrimination, racism, childbirth, fear of childbirth, PPD, women’s health, labor pain, mothers, side effects, labor support, estrogen, progesterone, oxytocin, inflammation, proinflammatory, cytokines, cortisol, CRH OR corticotropin-releasing hormone, ACTH OR adrenocorticotropic hormone, esketamine
Search Strings for Biological and Hormonal Factors	“pain” AND (“prenatal” OR “postpartum” OR “perinatal”) AND (estrogen OR progesterone OR oxytocin OR inflammation OR proinflammatory OR cytokines OR cortisol OR CRH OR corticotropin-releasing hormone OR ACTH OR adrenocorticotropic hormone OR esketamine)
Search Strings for Latinx Populations	“pain” AND (“prenatal” OR “postpartum” OR “perinatal”) AND (Latina OR Latinx OR Hispanic OR Latin American OR Central American OR South American OR Mexican OR Puerto Rican OR Salvadorian OR Cuban OR Dominican OR Guatemalan OR Colombian OR Honduran)
Search Strings for Asian Populations	“pain” AND (“prenatal” OR “postpartum” OR “perinatal”) AND (Asian OR Chinese OR Indian OR Korean OR Japanese OR Taiwanese OR Vietnamese OR Filipino)
Search Strings for Black/African/Caribbean Populations	“pain” AND (“prenatal” OR “postpartum” OR “perinatal”) AND (African American OR Black OR Sub-Saharan African OR Caribbean OR Nigerian OR Ethiopian OR Somali OR Ghanaian OR Jamaican OR Haitian OR Trinidadian and Tobagonian OR West Indian)
Database- Specific Search Examples	ScienceDirect: pain AND prenatal AND postpartum AND perinatal AND depression AND management Cochrane: pain AND prenatal OR postpartum OR perinatal AND depression AND labor Scopus: (pain) AND (prenatal OR postpartum OR perinatal) AND (depression) AND (risk)

After completing the searches, all articles were downloaded and uploaded to Rayyan, a tool designed to assist with literature searches and systematic reviews. The platform was used to identify and remove duplicates based on Title, Author(s), DOI, Year, Pages, and similarity percentage across articles. After eliminating duplicates, titles and abstracts were reviewed for inclusion. A third author conducted an additional assessment of the titles and abstracts to ensure that the initial reviewers did not overlook any relevant studies. Studies that did not meet the inclusion criteria, including those conducted outside the United States and in languages other than English, were excluded. Records that met the inclusion criteria were entered into an Excel spreadsheet and cross-referenced by each reviewer to determine whether to include or exclude them. Additionally, each reviewer conducted a reverse search, examining the studies and references cited in the articles. Any discrepancies were resolved by involving a third reviewer. Subsequently, a full-text review was conducted to identify the studies for inclusion. The three authors reached a consensus on which studies to include in this review. A PRISMA figure was generated to display the flow process.

This review did not involve human subjects; therefore, it did not require ethical approval from an institutional review board.

## Results

**Fig. 1:**
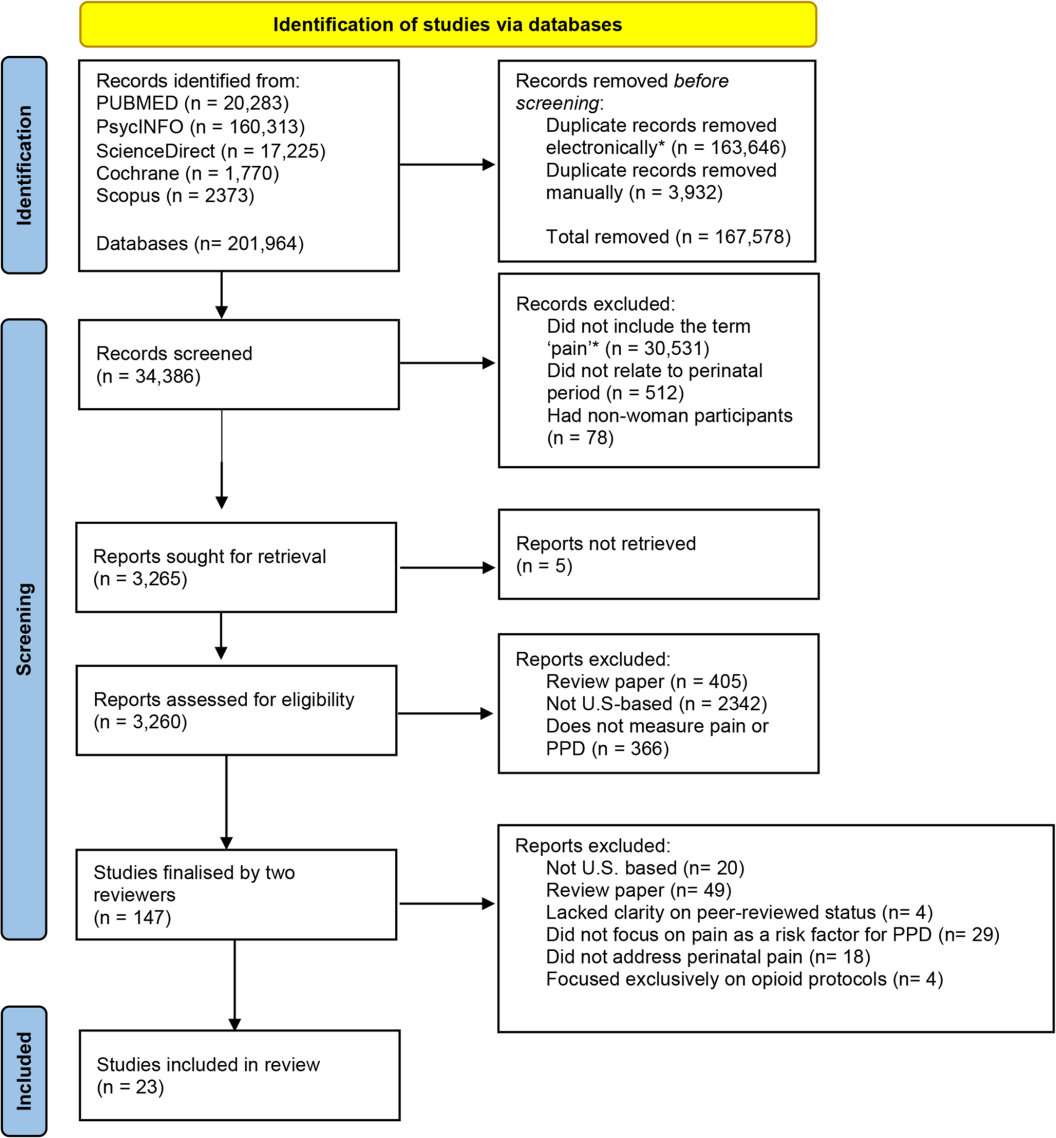
*Using Rayyan. PRIMSA Flowchart was generated using Page et al. [37]

Figure 1 shows the results from the literature search. The 23 articles selected for inclusion were categorized into seven pain-related risk factors: prenatal mental health, cesarean delivery, analgesics during and after childbirth, pain management beliefs and practices, discrimination in pain management, patients’ pain management preferences, and other pain-related factors (see Table 2).

**Table 2:** Summary of studies included in the critical review

Author(s)	Racial and Ethnic Composition	Subgroup analysis	Pain measure(s)	Pain-related factors
Mehdiratta et al. [11]	Caucasian/White participants (38.2%), Black/African American (38.4%), Asian (6.2%), and Others (17.2%).	No	Weighted Area Under the Curve (AUC) of verbal pain scores, measured on a 11-point (0 for no pain to 10 worst pain) scale during the irst 48 h after cesarean delivery.	● Pre-existing anxiety ● Repeat cesarean ● Analgesia: Use of intraoperative IV ketamine or fentanyl ● Other pain-related factors: Pre-existing chronic pain, current tobacco use, Black race, and not having public/private insurance.
Poehlmann et al. [12]	White sample (72.9%), with Black/African American (8.7%), Asian (8.9%), and other/unknown (1.0%) Hispanic ethnicity (8.3%)	Yes	Self-reported average pain scores (0–10 Numeric Rating Scale; NRS) during the first 24 h post-cesarean delivery.	● Depression and anxiety ● Cesarean delivery: Unscheduled or emergency cesarean delivery ● Other pain-related factors: Black racial identity
Sudhof et al. [13]	Non-Hispanic Whites (50.4%), Non-Hispanic Asians (11.7%), Non-Hispanic Blacks (14.6%), Hispanics (7.8%), “Other” (10.1%), and Unknown (4.3%)	No	Mean postpartum pain score (0–10 NRS) assessed during hospitalization and postpartum day 1 and 2 for vaginal and 1 and 4 for cesarean deliveries.	● Cesarean delivery and history of prenatal depression
Lim et al. [14]	Among those who did receive an epidural: 80% White, 16.4% Black, 3.6% American Indian, 7.3% Asian, and 1.8% “Other”; 7.4% of the epidural group were Hispanic/Latino.	No	Labor pain intensity and unpleasantness on a Visual Analog Scale (0 “no intensity at all” or “no unpleasantness at all” to 100 “the most intense pain I can imagine” or “the most unpleasant pain I can imagine”), Pain Treatment Satisfaction Scale Patient Treatment Satisfaction Scale [15] and Pain expectations on a 5-point Likert Scale (1 = greatly exceeds my expectations to 5 = does not meet my expectations at all)	● History of depression and anxiety were not predictive
Xu and Sampson [16]	54.5% Non-Hispanic White, 23.1% Hispanic, 15.3% Non-Hispanic Black, and 6.4% “Other”	No	Self-reported pain interference with routine activities within 2 months postpartum (5-point Likert scale: 1 = not at all to 5 = extremely)	● Cesarean delivery ● Discrimination in pain management: Holding back from communicating with providers, interaction between holding back and perceived discrimination, and holding back and low maternal care quality ● Other pain-related factors: Hospital stay and non-use of pain medication
Ende et al. [17]	59.2% White, 15.1% Black, 11% Hispanic, 7.5% Asian/Pacific Islander, 1.1% “Other,” and 5.8% had an Unknown racial/ethnic designation.	No	Maximum postpartum pain score on an 11-point rating scale on the day of surgery, during hospitalization, discharge, and 1–2 weeks post-discharge	● Intrapartum cesarean
Dinis et al. [18]	Of 170 participants across both groups, 50% were African American, 37.1% were Hispanic, 9.4% were White, 2.4% were Asian, and 1.2% were “Other”	No	Visual Analog Scale (0 no pain to 100 worst pain) 2–4 weeks postpartum and secondary outcome 5-point Likert scale (0 = no pain to 10 = worst pain)	● Analgesia: non opioids vs. opioids
Fowler et al. [19]	African Americans (2.5%), Asians (33.3%), Caucasians (41%), Pacific Islanders (1.2%), and “Other” (21.8%)	No	Numeric rating scores (0–10) 12 weeks after cesarean delivery.	● Analgesia: Gabapentin compared to placebo
Katz et al. [20]	Caucasian (56%), African American (9.6%), Asian (13.4%), Hispanic/Latino (14.4%), “Other” (9.8%), and missing < 1%	No	Verbal Pain Score (0–10 scale) at 24 h and 1 week postpartum	● Analgesia: preservative-free morphine or saline
O’Connor, et al. [21]	Race/ethnicity not reported.	No	NRS at 12–24 and 36–48 h post-cesarean.	● Analgesia: Spinal anesthesia with morphine among patients on buprenorphine
Loomis et al. [22]	92.4% Non-Hispanic, 6.5% Hispanic; 84.8% White, 7.6% Asian, 3.3% Black or African American, and 1.3% “Other”	No	Survey responses about pain score interpretation and medication practices	● Pain management beliefs and practices: “routine habit” and “patient preferences” pain-score threshold, cesarean delivery, and discharge counseling
Downs et al. [23]	Race/ethnicity not reported.	No	Qualitative provider-reported pain management practices	● Pain management beliefs and practices: Obstetricians’ clinical judgment, pain-score threshold, opioid reservation, and physical activity
Mackeen et al. [24]	Race/ethnicity not reported.	No	Qualitative interviews with obstetricians about prescribing practices	● Pain management beliefs and practices: Clinical and patient-centered pain prescription, consider social and contextual factors, and non-opioid first-line treatments
Kroll et al. [25]	53% Non-Hispanic White, 17% Non-Hispanic Black, 3% Hispanic/Latinx, 8% Asian American and Pacific Islander, 8% “Other,” and 11% Multiracial participants	No	Qualitative responses on patient pain and behavior during childbirth	● Pain management beliefs and practices: Preference for patients who do not complain, and blame patients for refusing analgesics
Nowakowski et al. [26]	Patients: Non-Hispanic White; Providers: race/ethnicity not reported	No	Mixed methods: qualitative interviews and provider survey ratings	● Pain management beliefs and practices: bias against patients with opioid use disorder, non-opioid preference, and collaborate with providers on pain medication administration
Fielding-Singh and Dmowska [27]	35% White, 24% Black, 26% Latina, and 15% Asian	No	Qualitative interviews on pain experiences during and after childbirth	● Discrimination in pain management: patients’ pain is dismissed, and leaving many feeling overlooked
Hoang et al. [28]	49% African American/Black; 24% Latina; 20% Asian/Asian Pacific Islander; 5% Multiracial; 2% Indigenous women	No	Qualitative interviews on provider communication and pain care	● Discrimination in pain management: patients’ pain is dismissed, and racism
Xiong et al. [29]	25 Hmong women from across the U.S.	No	Qualitative interviews on pain care and cultural context	● Discrimination in pain management: No pain discussions and cultural needs are unmet
Pauley et al. [30]	69% Non-Hispanic/Latino 31% Hispanic/Latino	No	Qualitative interviews on postpartum pain experiences and strategies	● Patients’ pain management preferences: Prescribed medications and behavioral pain management strategies (BPMS)
Badrelin et al. [31]	40.8% Non-Hispanic Black, 40.8% Hispanic, 6.1% Hispanic Black, 10.2% Non-Hispanic White, and 2%Asian	No	Qualitative interviews on postpartum pain experiences and strategies	● Patients’ pain management preferences: Prescribed medications and BPMS, some preference for opioids, but felt stigmatized, and preference for individualized care
Stump et al. [32]	72% White, 12% African American, 7% Asian, 9% “Other”/Multiracial, and 18% Hispanic/Latino.	No	Brief Pain Inventory short form [33] on day 7 (0 no pain to 10 pain as bad as you can imagine)	● Patients’ pain management preferences: Pain not predictive of obstetric recovery
Pham et al. [34]	65% non-Hispanic White, 16.1% non-Hispanic Black, 10.2% Hispanic, 2.9% Asian, 5.8% “Other”/Unknown	No	0–10 NRS (current and average pain), pain intensity on the Short Form McGill Pain Questionnaire- 2 [35]	● Other pain-related factors: cerebrospinal fluid and plasma β-Endorphin (BE) levels predictive
Babazade et al. [36]	Race/ethnicity not reported.	No	11-point NRS (Average pain scores over hospital stay)	● Other pain-related factors: Length of hospital stay and postpartum depression

## Prenatal Mental Health

Three retrospective studies [11–13] and one prospective study [14] examined the association between prenatal mental health and postpartum pain in patients undergoing a cesarean delivery. Sudhof et al.‘s cohort study of 6,094 racially and ethnically diverse patients found that those with a cesarean delivery and a history of antenatal depression experienced higher postpartum pain [13]. These findings remained significant even when accounting for substance use, psychiatric disorders, medication, and demographic factors [13]. Poehlmann and colleagues conducted multivariate regressions in a sample of 2,228 patients who underwent cesarean deliveries and found that racially and ethnically diverse women with depression (*p* = .001) and anxiety reported significantly higher mean pain ratings compared to those without (*p* < .001) [12]. Mehdiratta et al. conducted a study involving 1,899 patients and showed that when controlling for demographic characteristics and intraoperative factors, pre-existing anxiety (*p* = .006) was significantly associated with post-operative pain severity [11]. In contrast, in supplemental analyses, Lim et al. reported that a history of depression or anxiety did not significantly predict maximum pain intensity (*p* = .53) or pain unpleasantness (*p* = .41) six weeks postpartum [14]. This was based on a prospective observational study of 55 diverse patients who received or planned to receive labor epidural analgesia.

## Cesarean Delivery

Four studies [11, 12, 16, 17] examined the association between cesarean deliveries and postpartum pain using different designs. Xu and Sampson assessed 34 risk factors for postpartum pain in a racially and ethnically diverse sample of 2,461 English-speaking women who completed the Listening to Mothers III online survey in 2012 [16]. Results from machine learning indicated that cesarean delivery was a predictor of postpartum pain. Others found that specific aspects of a cesarean delivery are important.

Poehlmann et al. found that women who had unscheduled or emergency cesarean deliveries reported significantly higher pain ratings than those with scheduled cesarean deliveries, with p-values of 0.046 and less than 0.001, respectively [12]. Mehdiratta et al. found that repeat cesarean delivery increased the odds of pain severity (95% CI 1.18–2.02, *p* =.002) [11]. Ende and colleagues analyzed a cohort of 720 patients who underwent cesarean deliveries, with 392 experiencing labor beforehand and 328 not [17]. Their findings showed that patients who had an intrapartum cesarean delivery reported higher pain ratings on the day of delivery compared to those who did not, after adjusting for factors such as maternal age and race (average pain difference: 0.91, 95% CI 0.52–1.30, *p* < .0001).

## Analgesics During and After Childbirth

Five studies [11, 18–21] examined the differences in pain ratings by labor analgesics, with some notable observed differences. Mehdiratta and colleagues found that patients who received intraoperative fentanyl IV or ketamine IV had higher odds of severe pain after cesarean delivery compared to those who did not receive these interventions (95% CI 1.21–2.01, *p* < .0001) [11].

A randomized controlled trial involving 149 women who underwent cesarean deliveries was conducted by Dinis et al. [18]. Participants were randomly assigned to receive either outpatient non-opioid analgesics (ibuprofen combined with acetaminophen) or opioid treatment (ibuprofen plus hydrocodone-acetaminophen). Eighty-five women were placed in the non-opioid group, while the other 85 were assigned to the opioid group. The results from the ordinal logistic regressions controlling for intervention group and repeated cesarean showed that the non-opioid group had lower mean pain scores at 2 to 4 weeks after the procedure compared to the opioid treatment group, with an adjusted mean difference of 4.89 and a 95% confidence interval of −2.1 to 11.9 mm.

O’Connor et al. conducted a retrospective cohort study involving 92 women who underwent cesarean delivery [21]. This group included 46 women with opioid use disorder (OUD) who were treated with buprenorphine, matched with 46 control subjects within a five-year age range, primary insurance, and indication for a cesarean delivery. After adjusting for anesthesia type, smoking status, and buprenorphine dose in patients with OUD, the study found no significant differences in pain scores during hospitalization. However, among the patients on buprenorphine, those who received spinal anesthesia with morphine reported lower average pain levels compared to those on buprenorphine who received other forms of anesthesia (such as epidural, spinal anesthesia without morphine, or general anesthesia) (*p* = .01). Further analysis revealed that the mean pain scores over time for patients who received spinal anesthesia with morphine and were treated with buprenorphine were not significantly different from those in the control group [21].

Katz et al. conducted a trial with 157 diverse patients scheduled for vaginal delivery [20]. Within an hour of delivery, participants received either preservative-free morphine or saline via epidural. Results from the Mann-Whitney U tests showed significantly lower median pain scores 24 h post-delivery in the morphine group (2.0, IQR: 1–4) compared to the placebo group (3.0, IQR: 1.5–5.0), with a statistically significant difference (*p* = .043).

Fowler et al. conducted a double-blind, placebo-controlled trial involving 78 diverse patients who underwent cesarean delivery and were monitored for 12 weeks [19]. Participants were assigned to either a placebo or a gabapentin group to evaluate opioid cessation in the first week. Results from the Kaplan-Meier survival analysis showed that Gabapentin, aimed at aiding those with severe postpartum pain, showed no significant differences in pain ratings (*p* = .56), time to opioid cessation (*p* = .65), pain resolution (*p* = .20), or recovery (*p* = .37) among the 70 participants who completed the study.

## Pain Management Beliefs and Practices

Five studies [22–26] interviewed and surveyed nurses, obstetricians, and medical residents regarding their beliefs and practices related to pain management during and after childbirth. Loomis et al. surveyed 92 predominantly White postpartum bedside nurses. They found that 87% reported that patient-reported pain scores influenced the prescriptions for pain medication [22]. Factors such as “routine habit” and “patient preference” influenced dosing for 71% and 70% of respondents, respectively. A pain score of 7 or higher generally indicates the need for opioids. Notably, 95% of healthcare providers felt that most cesarean deliveries require pain management, though treatments mainly recommended were ibuprofen, perineal ice packs, and acetaminophen, with opioids rarely suggested. All providers in the study reported discussing pain management options with postpartum patients at discharge [22]. A qualitative study by Downs et al. involving 38 obstetricians and maternal-fetal medicine physicians found that 71% relied on clinical insights over regulatory guidelines for postpartum pain management [23]. Similarly to Loomis et al., 63% advocated for non-pharmacological techniques, when possible, while 89% endorsed using medications like acetaminophen for mild to moderate pain [22]. Opioids were considered necessary mainly for severe cases and days after a cesarean delivery. Additionally, 69% recommended physical activity for pain management, depending on the severity of the pain [23].

Mackeen and colleagues’ interviews with 38 obstetricians revealed that while all providers preferred prescription medication for pain, there were inconsistent practices in pain medication management [24]. Participants had different approaches to specific analgesics and the number of pills prescribed, and 79% reported not providing refills. Providers employed both clinical and patient-centered approaches when prescribing opioids, considering factors like patients’ pain reports and social circumstances. However, patient-centered factors were secondary to considerations like drug-seeking behaviors. Although, clinical advice and counseling were common, many providers preferred discussing non-opioid options first to reduce opioid reliance after childbirth [24].

In contrast to the patient-centered approach reported in the studies above, Kroll et al. found that 36 racially and ethnically diverse obstetrics and gynecology residents held positive views of women who were quiet and cooperative during childbirth, often labeling those who endured pain as heroic [25]. Qualitative data indicated that labor pain could complicate patient care. When patients refused analgesics like epidurals, their pain was seen as a result of their own choices rather than provider neglect. Reports on pain experiences varied by race, ethnicity, and income, suggesting that demographic factors influenced residents’ perceptions of patients’ pain responses during labor [25].

Lastly, a mixed-methods study by Nowakowski et al. involving 17 perinatal non-Hispanic White patients with opioid use disorder (OUD) and 15 healthcare providers found that providers reported that obstetric patients with OUD on buprenorphine or methadone experience worse pain (*p* = .003) and require more pain medication than those without OUD (*p* = .06) [26]. Providers preferred non-opioid analgesics (*p* = .003), nitrous oxide (*p* = .02), and complementary medicines (e.g., massage therapy, music therapy, and relaxation techniques) (*p* = .05) over opioids, while patients felt their opioid history did not impact their pain but expressed concerns about increased pain sensitivity. Providers recommended collaborating with postpartum nurses to enhance pain management, but nurses indicated they had a limited understanding of OUD-specific pain management [26].

### Discrimination in Pain Management

The following four studies provide context for patients’ pain-related experiences [16, 27–29]. A qualitative study by Fielding-Singh and Dmowska involving 46 racially and ethnically diverse women, found that participants reported feeling dismissed by healthcare providers regarding their pain complaints during and after childbirth, leaving many feeling overlooked [27]. Similarly, Hoang et al. studied Black, Indigenous, and other People of Color perinatal women [28]. They found that many Black and Latina participants felt their healthcare providers withheld information and ignored their pain management concerns. This dismissal led them to feel that their pain was overlooked because of their racial backgrounds. Additionally, a qualitative study by Xiong et al. involving 25 Hmong women highlighted the lack of discussion on the experiences of these less studied patients [29]. Participants shared their perinatal experiences, noting concerns about inadequate conversations and informed care regarding pain management during and after childbirth, which made them feel uninformed and unsupported. Participants reported insufficient discussions about their cultural preferences and the challenges of balancing cultural and Western practices, which led to unexpected decisions, such as unplanned cesarean deliveries. Finally, Xu and Sampson found that patients who limited information sharing with healthcare providers when used in the model alone and when interacted with perceived discrimination during their childbirth hospital stay were strong predictors of postpartum pain [16]. Additionally, the interaction between limiting shared information and low maternity care scores predicted pain scores [16].

## Patients’ Pain Management Preferences

Four studies explored preferences for managing postpartum pain, essential for identifying potential mismatches between healthcare providers’ practices and patients’ beliefs [26, 30–32]. Nowakowski and colleagues conducted a mixed-methods study involving perinatal patients with OUD and healthcare providers [26]. The study revealed that while patients generally preferred to avoid using opioids for pain management during and after childbirth—due to concerns about relapse and their anticipated ability to tolerate pain—some still desired options for managing severe pain, particularly after cesarean sections. Furthermore, patients expressed interest in analgesics like acetaminophen and gabapentin, as well as non-pharmacological options such as music therapy, relaxation techniques, and massage therapy. A qualitative study involving 32 racially and ethnically diverse individuals who had given birth in the past six months found that most participants (78%) used a combination of prescribed pain medications and behavioral pain management strategies (BPMS), including physical activity, massage, and relaxation [30]. These individuals reported that both the medication and BPMS were effective in managing their pain and contributed to quicker recovery. In a prospective qualitative study of English and Spanish-speaking patients’ experiences with post-cesarean pain management, Badreldin et al. also discovered that patients preferred using a mix of analgesics and BPMS, with several participants acknowledging the benefits of opioids [31]. However, some individuals felt stigmatized when requesting opioids. Another qualitative study that included 59 racially and ethnically diverse patients who had undergone cesarean deliveries revealed that participants perceived their pain as unique, suggesting that a one-size-fits-all approach to pain management is neither practical nor feasible. Stump et al. conducted a study with 551 predominantly White participants planning vaginal deliveries [32]. Patients reported an average pain score of 2.6 (SD = 2.1). Their adjusted models, which accounted for factors such as anxiety history and neonatal intensive care admissions, revealed no correlation between the Obstetric Quality Recovery-10 items and postpartum pain levels on day of discharge. However, they did not consider individual factors related to the Obstetric Quality Recovery-10 measure, such as patient experiences.

## Other Pain-Related Factors

Five studies identified additional risk factors for post-childbirth pain beyond the previously discussed ones [11, 12, 16, 34, 36]. In retrospective studies of cesarean delivery patients, significant risk factors included tobacco use, Black or African American ethnicity, gravidity of four or more, a history of chronic pain, and longer hospital stays [11, 12, 16, 36]. A lack of analgesia also contributed to post-childbirth pain. One hormone-related study found a significant association between plasma levels of endogenous opioid β-Endorphin (BE) and pain reported two weeks after cesarean delivery (*p* < .01) [34]. In this prospective study of 136 women without opioid use or chronic pain, results from the hierarchical linear regressions adjusting for confounders (e.g., age, BMI, and race) showed that cerebrospinal fluid BE levels were positively associated with pain intensity 48 h post-surgery (*p* = .05). Additionally, plasma BE levels predicted postpartum pain at 48 h (*p* = .02) and two weeks (*p* < .01).

## Discussion

Post-childbirth pain is linked to an increased risk of postpartum depression. Our review of the recent literature highlights various interrelated factors contributing to heightened pain after childbirth. Key findings suggest that psychological vulnerability, particularly histories of depression and anxiety, plays a significant role in increased postpartum pain, especially among cesarean delivery patients. For example, Sudhof et al. [13] found that depression during pregnancy correlates with higher postpartum pain in cesarean section cases. Similarly, Poehlmann et al. [12] and Mehdiratta et al. [11] found that demographic factors and mental health histories increased the odds of post-cesarean pain severity.

Though none of the investigators conducted subgroup analyses due to small sample sizes, the findings remain relevant for racial and ethnic minorities. One retrospective study indicated that Black and Puerto Rican birthing people are at an elevated risk of depression during pregnancy compared to White individuals, experiencing more moderate to severe depressive symptoms. This aligns with previous research showing Black individuals face a greater risk and symptom severity in pregnancy [38, 39].

This review also showed that while a cesarean delivery, including repeat cesareans, is a significant risk factor for postpartum pain [11, 16], the complexities of this delivery must be considered. For example, Poehlmann et al. [12] found that individuals with an unscheduled or emergency cesarean delivery experienced significantly higher postpartum pain than those with a scheduled cesarean delivery. Black and Asian individuals are more likely to undergo unplanned cesareans, contributing to disparities in outcomes [40]. These trends are particularly concerning because racial and ethnic individuals giving birth have a higher likelihood of undergoing cesarean deliveries compared to White individuals [40, 41]. Furthermore, Ende et al. [17] showed that going into labor before a cesarean delivery also increases the risk of elevated postpartum pain, highlighting the importance of potentially intersecting risk factors for post-operative pain. These trends are particularly concerning because racial and ethnic individuals giving birth have a higher likelihood of undergoing cesarean deliveries compared to White individuals [40, 41]. Additionally, research has shown that Black and Asian birthing individuals face an increased risk of unplanned cesarean deliveries [40].

Our review also revealed that pain management is a crucial factor in addressing pain-related issues. Morphine was effective at reducing pain in two studies [20, 21], but gabapentin [19] and intraoperative fentanyl IV or ketamine IV were associated with increased post-operative pain severity [11]. Dinis et al. [18] found that patients who delivered by cesarean and received non-opioid analgesics reported lower postpartum pain scores compared to those who had. O’Connor [21] adjusted for anesthesia type, smoking status, and buprenorphine dose. However, Katz et al. [20] and Fowler et al. [19] did not control for potential covariates or confounding variables, underscoring the bivariate nature of the results. Still, while certain analgesics may not be effective at reducing the risk for post-childbirth pain, so are providers’ practices and beliefs. This review underscores the role providers play in managing pain after childbirth, whether it is through a cesarean or vaginal delivery. Our review revealed that most studies of healthcare providers (e.g., nurses and physicians) indicate that they rely on patient reports and clinical assessments to manage birthing people’s pain [22–24]. The studies we reviewed also demonstrated that providers manage postpartum pain based on the mode of childbirth and patient circumstances, in consultation with patients [22, 23, 26]. However, the data from patients suggest otherwise. We found that racial and ethnic minority birthing people feel discriminated against, ignored, and their pain is dismissed [27–29]. Additionally, Xu and Sampson found that withholding information from providers was a robust risk factor for postpartum pain, including when it interacted with perceived discrimination and maternal care quality [16].

These findings underscore the need for shared decision-making and responsive and culturally sensitive patient care. Our findings indicate that patients want to be heard and have options for treating post-childbirth pain, particularly after a cesarean delivery. Two studies showed that patients need individualized pain management [31], including medication and behavior strategies [30]. Two studies in our review showed that some providers recommend alternative approaches to pain medication [22, 26].

Culturally sensitive and individualized pain management approaches that combine medication and behavioral strategies are needed [26, 30, 31]. Patients and healthcare providers may share some beliefs regarding different approaches to managing pain after childbirth. However, it is essential to encourage these conversations in a safe and supportive healthcare environment. This environment should be responsive to patients’ needs and free from discrimination while valuing their experiences [22, 26].

This critical review highlights factors that increase the risk of post-childbirth pain, particularly among racial and ethnic minorities. These groups are less likely to receive adequate postpartum pain management and experience higher rates of postpartum depression [2, 9]. Although most studies reviewed included diverse samples, only one conducted subgroup analyses, and none had equal sample sizes across groups. Additionally, only one study included non-English speakers, emphasizing the need for greater diversity in future research.

## Limitations

This study identified seven interrelated pain-related risk factors across 23 studies focused on post-childbirth pain. However, several limitations need to be addressed. First, the review considered only studies conducted between 2020 and 2025, potentially overlooking additional relevant research. Second, it focused on peer-reviewed English-language studies, thereby excluding perspectives and experiences in studies published in languages other than English. Third, most of the studies focused on cesarean deliveries, limiting our ability to assess pain-related factors among vaginal deliveries. Fourth, there was heterogeneity across pain measures and the timing of those measures, making it impossible to compare across studies. Fifth, the study did not establish a connection between postpartum pain and depression, suggesting the need for further exploration of both direct and indirect associations. Our study focused exclusively on U.S.-based research, which may limit the generalizability of findings to other contexts. Lastly, the studies reviewed had several limitations, including reliance on retrospective and self-reported data, a lack of diversity in the sample populations, a limited number of cities or regions represented, and potential biases such as recall bias or social desirability effects. Additionally, there were unmeasured confounding factors, and the nature of the data and analyses restricted the ability to make causal interpretations, impacting the findings’ generalizability. To enhance our understanding of the complex relationship between postpartum pain and the risk of postpartum depression among racial and ethnic minorities, future studies should focus on including diverse samples, employing prospective study designs, and utilizing multivariate models that account for confounding variables.

## Conclusions

A variety of interconnected factors influence post-childbirth pain, yet most studies fail to consider these varying elements together. This critical review identifies several related factors that contribute to post-childbirth pain, particularly among racial and ethnic minorities. Our findings can help inform a biopsychosocial model that outlines the interconnected factors increasing the risk of postpartum pain, which may also elevate the risk of postpartum depression among these groups. While psychological vulnerability is linked to higher levels of post-childbirth pain, these individual factors are further complicated by the mode of delivery, which is influenced by pain management strategies and healthcare provider beliefs. Additionally, discrimination and feelings of alienation among patients complicate the post-childbirth pain experiences of racial and ethnic minority birthing individuals. Together, these interconnected factors emphasize the need for individualized, equitable, and compassionate postpartum care. Such an approach would enhance postpartum care quality, improve pain management protocols, and reduce the risk of postpartum depression.

## Key References

- Badreldin N, DiTosto JD, Leziak K, Niznik CM, & Yee LM. Understanding the Postpartum Cesarean Pain Experience Among Individuals With Publicly Funded Insurance: A Qualitative Investigation. Journal of Midwifery & Women's Health. 2024*:*6;9(1):136-43. https://doi.org/10.1111/jmwh.13540.
  - ○ The data from from this patient-centered study highlight the experiences of racially and ethnically diverse birthing individuals after a cesarean delivery, clarifying their preferences for post-childbirth pain management and the barriers they face.
- Dinis J, Soto E, Pedroza C, Chauhan SP, Blackwell S, & Sibai B. Nonopioid Versus Opioid Analgesia after Hospital Discharge Following Cesarean Delivery: A Randomized Equivalence Trial. American Journal of Obstetrics and Gynecology. 2020*;*222(5):e481-88.
  - ○ This randomized clinical trial involving diverse birthing people showed that non-opioid analgesics effectively relieve pain after cesarean deliveries.
- Loomis BR, Yee LM, Hayes L, & Badreldin N. Nurses' Perspectives on Postpartum Pain Management. Women's Health Reports. 2022;3(1):318-25.
  - ○ Examining racially and ethnically diverse front-line healthcare providers enhances our understanding of the various psychosocial and medical factors they consider when treating pain in birthing individuals.
- Poehlmann JR, Stowe ZN, Godecker A, Xiong PT, Broman AT, & Antony KM. The impact of preexisting maternal anxiety on pain and opioid use following cesarean delivery: a retrospective cohort study. American Journal of Obstetrics & Gynecology MFM. 2022;4(3):100576.
  - ○ This study of racially and ethnically diverse birthing people provided key information on the critical role peripartum experiences have on pain immediately after a cesarean delivery, including among Black individuals.
- Xu W, & Sampson M. Prenatal and Childbirth Risk Factors of Postpartum Pain and Depression: A Machine Learning Approach. Maternal and Child Health Journal. 2023;27(2):286-96. https://doi.org/10.1007/s10995-022-03532-0.
  - ○ The results of this innovative secondary data analysis highlight the importance of exploring and addressing patients’ experiences during childbirth and recovery, particularly regarding negative behaviors by healthcare providers.

## Data Availability

The data will be provided upon request from the lead author.

## References

[1] Van Niel MS, Payne JL. Perinatal depression: A review. Cleve Clin J Med. 2020;87(5):273–7. 32357982 10.3949/ccjm.87a.19054

[2] Khadka N, et al. Trends in postpartum depression by race, ethnicity, and prepregnancy body mass index. JAMA Netw Open. 2024;7(11):e2446486-2446486. 39565621 10.1001/jamanetworkopen.2024.46486 PMC11579791

[3] Swift A. Understanding pain and the human body’s response to it. Nurs Times. 2018;114(3):22–6.

[4] Gaudet C, Wen SW, Walker MC. Chronic perinatal pain as a risk factor for postpartum depression symptoms in Canadian women. Can J Public Health. 2013;104(5):E375. 24183178 10.17269/cjph.104.4029 PMC6974069

[5] Eisenach JC, et al. Severity of acute pain after childbirth, but not type of delivery, predicts persistent pain and postpartum depression. Pain. 2008;140(1):87–94. 18818022 10.1016/j.pain.2008.07.011 PMC2605246

[6] Weis CA, et al. Prevalence of low back pain, pelvic girdle pain, and combination pain in a pregnant Ontario population. J Obstet Gynaecol Can. 2018;40(8):1038–43. 30103876 10.1016/j.jogc.2017.10.032

[7] Jin J, et al. Prevalence and risk factors for chronic pain following Cesarean section: a prospective study. BMC Anesthesiol. 2016;16(1):99. 27756207 10.1186/s12871-016-0270-6 PMC5069795

[8] Weeks F, et al. Are experiences of Racial discrimination associated with postpartum depressive symptoms? A multistate analysis of pregnancy risk assessment monitoring system data. J Women’s Health. 2022;31(2):158–66. 10.1089/jwh.2021.0426 PMC10941332 34967671

[9] Badreldin N, Grobman WA, Yee LM. Racial disparities in postpartum pain management. Obstet Gynecol. 2019;134(6):1147–53. 31764723 10.1097/AOG.0000000000003561 PMC6905121

[10] Johnson JD, et al. Racial and ethnic inequities in postpartum pain evaluation and management. Obstet Gynecol. 2019;134(6):1155–62. 31764724 10.1097/AOG.0000000000003505

[11] Mehdiratta J, et al. Patient and procedural risk factors for increased postoperative pain after Cesarean delivery under neuraxial anesthesia: a retrospective study. Int J Obstet Anesth. 2020;44:60–7. 32799069 10.1016/j.ijoa.2020.07.006

[12] Poehlmann JR, et al. The impact of preexisting maternal anxiety on pain and opioid use following Cesarean delivery: a retrospective cohort study. Am J Obstet Gynecol MFM. 2022;4(3):100576. 35114423 10.1016/j.ajogmf.2022.100576

[13] Sudhof LS, Gompers A, Hacker MR. Antepartum depressive symptoms are associated with significant postpartum opioid use. Am J Obstet Gynecol MFM, 2023. 5(8). 10.1016/j.ajogmf.2023.101009 PMC10524126 37156465

[14] Lim G, et al. Obstetric pain correlates with postpartum depression symptoms: a pilot prospective observational study. BMC Pregnancy Childbirth. 2020;20(1):1–14. 10.1186/s12884-020-02943-7 PMC7178606 32321455

[15] Evans CJ, Trudeau E, Mertzanis P, Marquis P, Pena BM, Wong J, et al. Development and validation of the pain treatment satisfaction scale (PTSS): a patient satisfaction questionnaire for use in patients with chronic or acute pain. Pain. 2004;112:254–66. 10.1016/j.pain.2004.09.005 15561380

[16] Xu W, Sampson M. Prenatal and childbirth risk factors of postpartum pain and depression: a machine learning approach. Matern Child Health J. 2023;27(2):286–96. 36526882 10.1007/s10995-022-03532-0

[17] Ende HB, et al. Labor prior to Cesarean delivery associated with higher post-discharge opioid consumption. PLoS One. 2021;16(7):e0253990. 34242277 10.1371/journal.pone.0253990 PMC8270408

[18] Dinis J, et al. Nonopioid versus opioid analgesia after hospital discharge following Cesarean delivery: a randomized equivalence trial. Am J Obstet Gynecol. 2020;222(5):488. e1-488. e8. 10.1016/j.ajog.2019.12.001 31816306

[19] Fowler C, et al. Outpatient treatment with Gabapentin in women with severe acute pain after Cesarean delivery is ineffective: a randomized, double-blind, placebo-controlled trial. Anesth Analgesia. 2023;136(6):1122–32. 10.1213/ANE.0000000000006429 37043404

[20] Katz D, et al. Impact of neuraxial preservative-free morphine in vaginal delivery on opiate consumption and recovery: a randomized control trial. Anesthesia & Analgesia. 2022:101213. 10.1213/ANE.0000000000006987 39028662

[21] O’Connor AB, et al. Peripartum and postpartum analgesia and pain in women prescribed buprenorphine for opioid use disorder who deliver by Cesarean section. Subst Abuse: Res Treat. 2022;16:11782218221107936. 10.1177/11782218221107936 PMC9218889 35754980

[22] Loomis BR, et al. Nurses’ perspectives on postpartum pain management. Women’s Health Rep. 2022;3(1):318–25. 10.1089/whr.2021.0104 PMC8994431 35415715

[23] Downs DS, et al. Obstetric physicians’ beliefs and knowledge on guidelines and screening tools to reduce opioid use after childbirth. Obstet Gynecol. 2021;137(2):325–33. 33416288 10.1097/AOG.0000000000004232 PMC10846479

[24] Mackeen AD, et al. Obstetricians’ prescribing practices for pain management after delivery. Pain Manage. 2022;12(5):645–52. 10.2217/pmt-2021-0101 PMC10015511 35289656

[25] Kroll C, et al. Cultivating the ideal obstetrical patient: how physicians-in-training describe pain associated with childbirth. Volume 312. Social Science & Medicine. 2022:115365. 10.1016/j.socscimed.2022.115365 36155358

[26] Nowakowski E, et al. Obstetric pain management for pregnant women with opioid use disorder: a qualitative and quantitative comparison of patient and provider perspectives (QUEST study). Addiction. 2023;118(6):1093–104. 36662775 10.1111/add.16134 PMC10175133

[27] Fielding-Singh P, Dmowska A. Obstetric gaslighting and the denial of mothers’ realities. Soc Sci Med. 2022;301:114938. 35395611 10.1016/j.socscimed.2022.114938 PMC9167791

[28] Hoang T-MH, et al. Experiences of Racial trauma among perinatal women of color in seeking healthcare services. Gen Hosp Psychiatry. 2023;84:60–6. 37393649 10.1016/j.genhosppsych.2023.06.015

[29] Xiong S, Yu Z, Lor M. Experiences of Hmong women in the perinatal period. Journal of Obstetric, Gynecologic & Neonatal Nursing. 2025. 10.1016/j.jogn.2025.03.001 40164227

[30] Pauley AM, et al. Women’s beliefs of pain after childbirth: critical insight for promoting behavioral strategies to regulate pain and reduce risks for maternal mortality. Patient Educ Couns. 2023;107:107570. 36410313 10.1016/j.pec.2022.11.012 PMC9789185

[31] Badreldin N, et al. Understanding the postpartum Cesarean pain experience among individuals with publicly funded insurance: A qualitative investigation. J Midwifery Women’s Health. 2024;69(1):136–43. 37394901 10.1111/jmwh.13540 PMC10758503

[32] Stump CM, et al. Association of inpatient postpartum recovery with patient-reported outcome measures following hospital discharge: a prospective cohort study. BMC Pregnancy Childbirth. 2024;24(1):618. 39342111 10.1186/s12884-024-06805-4 PMC11438177

[33] Poquet N, Lin C. The brief pain inventory (BPI). J Physiother. 2016;62(1):52. 26303366 10.1016/j.jphys.2015.07.001

[34] Pham A et al. Prospective evaluation of cerebrospinal fluid levels of β-Endorphin as a predictor of opioid use after scheduled cesarean delivery. Research Square. 2023: p. rs. 3. rs-3125641. 10.1186/s12884-025-08613-w PMC12859979 41462136

[35] Dworkin RH, et al. Development and initial validation of an expanded and revised version of the Short-form McGill pain questionnaire (SF-MPQ-2). Pain^®^. 2009;144(1–2):35–42. 19356853 10.1016/j.pain.2009.02.007

[36] Babazade R, et al. Acute postcesarean pain is associated with in-hospital exclusive breastfeeding, length of stay and post-partum depression. J Clin Anesth. 2020;62:109697. 31899076 10.1016/j.jclinane.2019.109697

[37] Page MJ et al. The PRISMA 2020 statement: an updated guideline for reporting systematic reviews. BMJ. 2021;372. 10.1136/bmj.n71 PMC8005924 33782057

[38] Sujan AC, et al. Racial and ethnic differences in perinatal depression and anxiety. J Affect Disord. 2023;334:297–301. 37156281 10.1016/j.jad.2023.04.123 PMC10234114

[39] Kelly-Taylor K, et al. Prenatal depression and symptom severity by maternal race and ethnicity. JAMA Netw Open. 2025;8(3):e250743–250743. 40080023 10.1001/jamanetworkopen.2025.0743 PMC11907315

[40] Williams A, et al. Mode of delivery and unplanned cesarean: differences in rates and indication by race, ethnicity, and sociodemographic characteristics. Am J Perinatol. 2024;41(07):834–41. 35235955 10.1055/a-1785-8843

[41] Okwandu IC, et al. Racial and ethnic disparities in Cesarean delivery and indications among Nulliparous, Term, Singleton, vertex women. J Racial Ethn Health Disparities. 2022;9(4):1161–71. 34254270 10.1007/s40615-021-01057-w PMC9249704

